# Bisphosphonates with high bone-resorption-capacity promote osteonecrosis of the jaw development after tooth extraction in mice

**DOI:** 10.1007/s00774-025-01608-9

**Published:** 2025-05-28

**Authors:** Ryuta Kubo, Rui Tajiri, Hibiki Yamada, Hideki Nakayama, Takeshi Miyamoto

**Affiliations:** 1 Faculity of Life Science, Department of Oral and Maxillofacial Surgery, Kumamoto University, 1-1-1 Honjo, Chuo-Ku, Kumamoto, 860-8556 Japan; 2 Faculty of Life Sciences, Department of Orthopedic Surgery, Kumamoto University, 1-1-1 Honjo, Chuo-Ku, Kumamoto, 860-8556 Japan

**Keywords:** Osteonecrosis of the jaw, Zoledronate, Alendronate, Ibandronate, Osteoclast

## Abstract

**Introduction:**

Medication-Related Osteonecrosis of the Jaw (MRONJ) is a condition marked by osteonecrosis of the jaw bone and other symptoms seen following invasive surgical procedures in patients administered bone-modifying agents. Once disease develops, a patient’s ADL levels are significantly compromised. However, the pathogenesis of this disease is not clearly understood.

Bisphosphonates (BPs) are bone resorption inhibitors commonly used to treat osteoporosis. Although not confirmed, it is generally believed that MRONJ risk is higher in the presence of injectable rather than oral formulations. Here, we assessed risk of developing ONJ in mice in the presence of 3 different BPs—zoledronate, ibandronate, or alendronate—that are administered clinically intravenously or via infusion.

**Materials and Methods:**

Eight-week-old wild-type mice were administered zoledronate, alendronate, ibandronate or PBS vehicle subcutaneously once a week for 2 weeks. Then the right first molars in the mandible were extracted. Six-weeks later, osteonecrosis development was analyzed by histochemistry.

**Results:**

Among mice administered BPs, mice treated with zoledronate exhibited the highest frequency of osteocytes exhibiting osteonecrosis. Bone mineral density was higher in mice receiving zoledronate, alendronate, or ibandronate than in PBS control mice, but effects of the 3 drugs were comparable. Moreover, formation of multi-nuclear osteoclasts in vitro was most strongly inhibited by zoledronate, followed by alendronate and ibandronate.

**Conclusion:**

Administration of BPs with high osteoclastogenesis inhibitory potential, such as zoledronate, increases risk of ONJ development after tooth extraction more than treatment with other agents tested, even at equivalent dosage.

## Introduction

Medication-related osteonecrosis of the jaw (MRONJ) is a treatment-refractory form of osteonecrosis of the jaw (ONJ) seen in patients treated with bone-modifying agents (BMAs) [1]. BMAs, such as bisphosphonates (BPs) and denosumab, are used to treat osteoporosis and metastatic bone tumors. Treatment of MRONJ, which was first reported by Marx et al. in 2003 [2], typically involves use of antibiotics, debridement, and surgical removal of necrotic bone [1, 3]. However, this approach is often ineffective, and the condition is known to significantly impact patients’ quality of life. In addition to BPs, ONJ has been reported in patients treated with bevacizumab [4], a targeted anti-cancer drug with anti-angiogenic effects. The etiology of ONJ is thought to be multifactorial, involving infection or inflammatory tooth disease as well as inhibition of bone remodeling resulting from use of bone resorption inhibitors [1]. However, mechanisms underlying the condition remain unknown. BMA treatment reportedly prevents skeletal-related events (SREs) such as bone destruction caused by metastatic bone tumors, suppresses hypercalcemia caused by bone destruction, maintains activities of daily living (ADL) and quality of life, and increases life expectancy[5, 6]. Therefore, doctors may be hesitant to discontinue BMA use in patients who have developed MRONJ, as it is critical to block its progression.

BMAs are also known to potently suppress bone remodeling, a outcome known as severely suppressed bone turnover (SSBT) [7–9], which turn promotes MRONJ development. However, administration of angiogenesis inhibitors increases MRONJ risk, even in the absence of SSBT status [10, 11]. That risk is also increased by steroid administration [12] and diabetes mellitus [13, 14], while infection can exacerbate the condition [15, 16]. Inflammation is a factor in MRONJ development, as analysis zoledronate-treated versus control mice shows that tooth extraction significantly worsens ONJ and increases inflammatory cytokine expression at the extraction site in zoledronate relative to control mice [17, 18]. Local and systemic inflammatory cytokine expression reportedly increases during extraction, and administration of anti-inflammatory drugs significantly suppresses ONJ onset in that mouse model [19]. MRONJ risk is also increased by exposure of the extraction site to oral bacteria after tooth extraction [20]. In fact, analysis of a mouse model of MRONJ indicates that antibiotic treatment significantly reduces ONJ development [19].

The number of osteoporosis patients continues to grow as the elderly population increases [21, 22]. Today, BPs are the most widely used drugs to treat osteoporosis [22], and BPs such as zoledronate, alendronate and ibandronate, have been developed and used in clinical practice. Because BPs are absorbed extremely inefficiently from the intestinal tract [23], injectable ibandronate and intravenous formulations of zoledronate and alendronate are now commercially available. These drugs can be effective at lower doses than those used for oral formulations. Moreover, although it is not confirmed experimentally, MRONJ risk is considered more of a concern for injectable compared to oral formulations. Because zoledronate, ibandronate, and alendronate are administered clinically at different dosages and with different dosing regimens, it is not clear which drug poses the greatest risk of MRONJ development at a given drug dosage or dosing regimen. These questions could be evaluated in mouse models of MRONJ, which have proven useful in enabling quantitative evaluation of MRONJ by analyzing the rate of formation of empty lacunae in jaw bones. Other experimental approaches potentially relevant to this analysis include in vitro systems enabling evaluation of osteoclast differentiation that were developed after the discovery of RANKL [24]. Prior to this, osteoclast differentiation was induced by adding active vitamin D3 or cytokines such as IL-6 to co-cultures of osteoclast precursor cells and osteoblastic cells [25]. Now, however, osteoclast differentiation can be analyzed without osteoblastic cells by assessing osteoclast progenitor cell activity in the presence of M-CSF and RANKL [26], Using these types of assays, the effects of various factors or small molecules on osteoclastogenesis can also be directly evaluated. For our purposes, these assays could be useful in determining whether drugs that impact the degree of MRONJ development in mouse models also might inhibit osteoclastogenesis in vitro.

In this study, we evaluated ONJ risk in a mouse model of tooth extraction using equivalent doses of zoledronate, alendronate, or ibandronate. When we assessed the frequency of empty lacunae in bone, which is a marker of necrosis and thus ONJ, that frequency was highest with zoledronate, followed by ibandronate, which was comparable to alendronate. The increase in bone mineral density with drug administration was also highest with zoledronate, followed by ibandronate and alendronate, although that difference was not significant. Zoledronate also more potently inhibited osteoclastogenesis in vitro than did ibandronate and alendronate. These results indicate that risk of developing ONJ differs among bisphosphonate drugs, and that patients administered agents that strongly inhibit osteoclastogenesis need care to prevent ONJ development.

## Results

### Administration of zoledronate, alendronate, or ibandronate before and after tooth extraction increases risk of ONJ development in the jaw bone

**Fig. 1:**
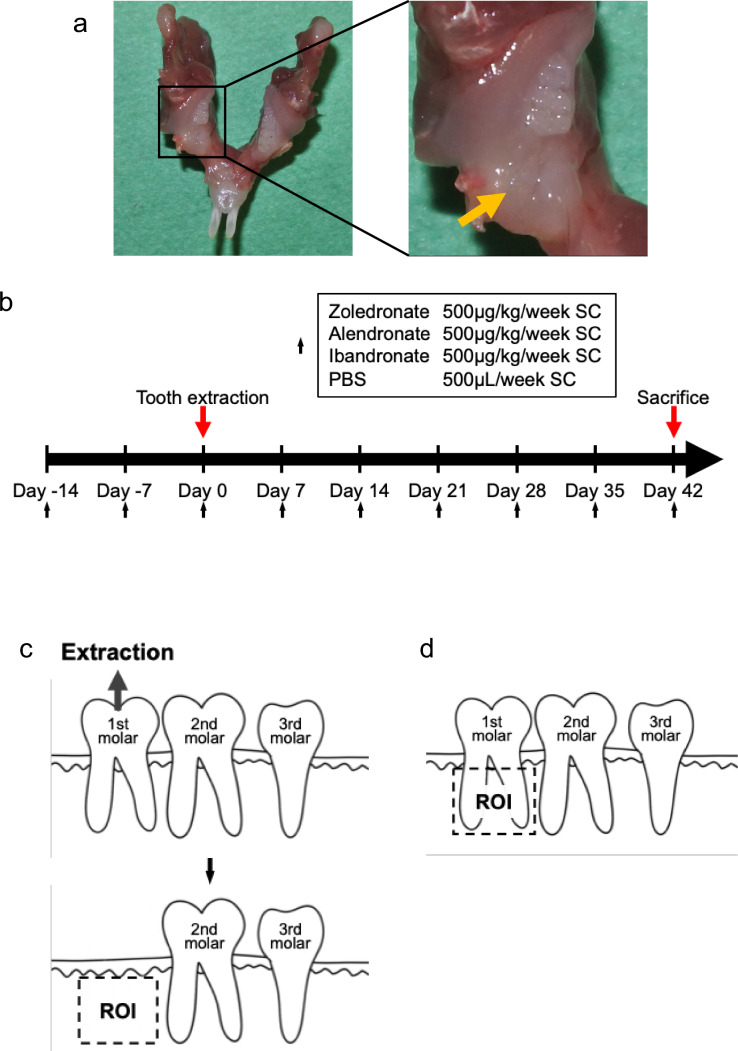
Establishment of ONJ model mice. **a** Timeline of ONJ model mouse creation. Zoledronate (ZOL), ibandronate (IBN), alendronate (ALN), or vehicle (PBS) were administered subcutaneously to 8-week-old female C57BL/6J wild-type mice. All treatments were administered at a dosage of 500 μg/kg per week. Two weeks later, the right first molar was extracted under anesthesia. Finally, mice were euthanized after 6 weeks. **b** Low (left) and high (right) magnification views of a representative mandible. The lower right first molar was extracted, and a bone palpable fistula had formed at the extraction site (yellow arrow). **c** and **d** Schemes demonstrate the location of the region of interest (ROI) for counting the percentage of empty lacunae in the extracted (**c**) and non- extracted side (**d**)

**Fig. 2:**
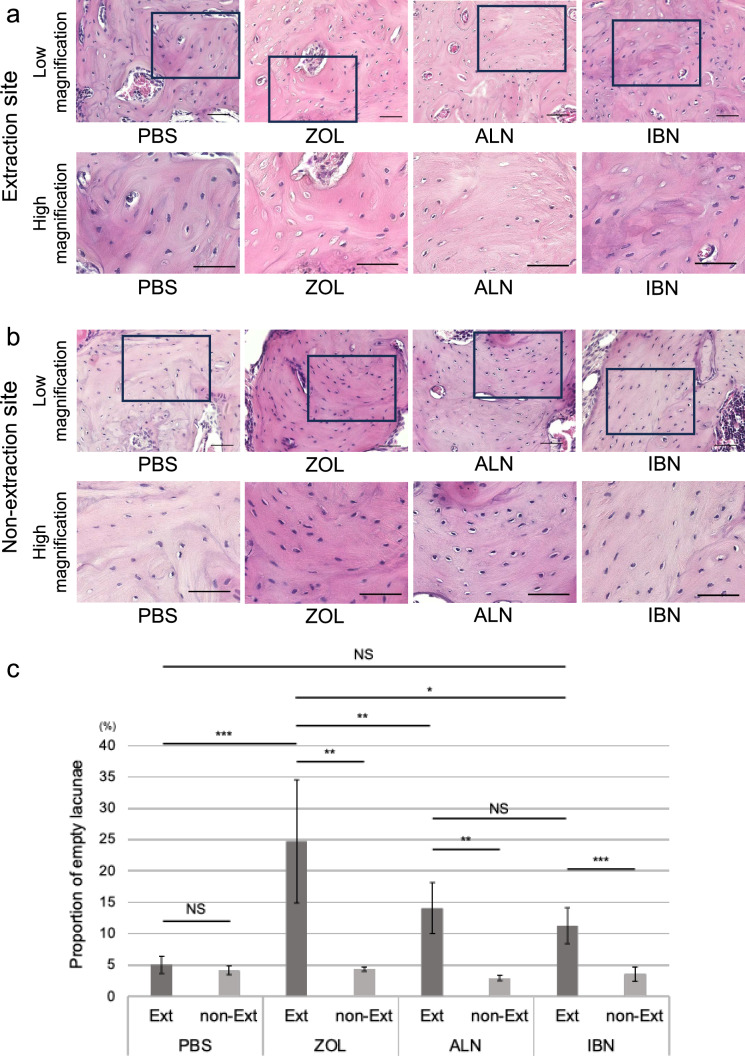
Zoledronate treatment increases the proportion of empty lacunae seen after tooth extraction in mice. **a** Histological evaluation of the tooth extraction site. Eight-week-old female wild-type mice were administered zoledronate (ZOL), ibandronate (IBN), alendronate (ALN), or vehicle (PBS) (500 μg/kg/week each) once a week for two weeks and then the first molars in the mandible were extracted, as shown in Fig. 1. Mice were then administered ZOL, IBN, ALN or PBS once a week for 6 more weeks. Mice were sacrificed when they were 16-weeks-old, and mandibles were removed, demineralized, paraffin-embedded, and sectioned for HE staining. Shown are low and high magnification images, as indicated. Boxed areas in upper panels are shown at higher magnification in corresponding lower panels. Scale bars = 50 μm. (**b** Histological evaluation of the non-extraction site. Model mice were created using the same protocols used in Fig. 2a, and sections obtained from the area of the first molar in the mandible at the contralateral tooth, which had not been extracted. Shown are low and high magnification images, as indicated. Scale bars = 50 μm. **c** Calculation of the mean relative proportion ± SD of empty to total lacunae assessed in jaw bone at the site of the first molar (n = 7 ~ 10, *p < 0.05; **p < 0.01; ***p < 0.001; NS, not significant, based on one-way ANOVA and the Mann-Whitney test)

Administration of intravenous zoledronate or alendronate or injectable ibandronate bisphosphonate preparations is considered a risk for MRONJ development [27, 28]. To compare effects of intravenous administration of these 3 drugs versus controls, we administered zoledronate, alendronate, ibandronate, or PBS to 8-week-old female mice at 500 μg/kg/week for 2 weeks once a week prior to extraction of the right first molars in the mandible and then once a week for 6 weeks after extraction (Fig. 1a and b). Then, when mice were 16 weeks-old, we evaluated ONJ frequency by calculating the percentage of empty lacunae among all osteocyte lacunae in jaw bones at the extraction site (Figs. 1c and 2a) and performed the same evaluation on the non-extraction site (Figs. 1d and 2b). ONJ frequency in the extracted jaw bone was greater than that in the non-extracted jaw bone in the case of all 3 drugs but was comparable in the PBS control group (Fig. 2c). On the other hand, in the BP group, in the presence of zoledronate, alendronate, or ibandronate, ONJ frequency in the extracted jaw bone was significantly higher than that in the non-extracted jaw bone (Fig. 2c). Zoledronate-treated mice showed a significantly higher ONJ frequency in the jaw bone than did alendronate- or ibandronate-treated mice, and there was no significant difference between the alendronate and ibandronate groups (Fig. 2c).

**Fig. 3:**
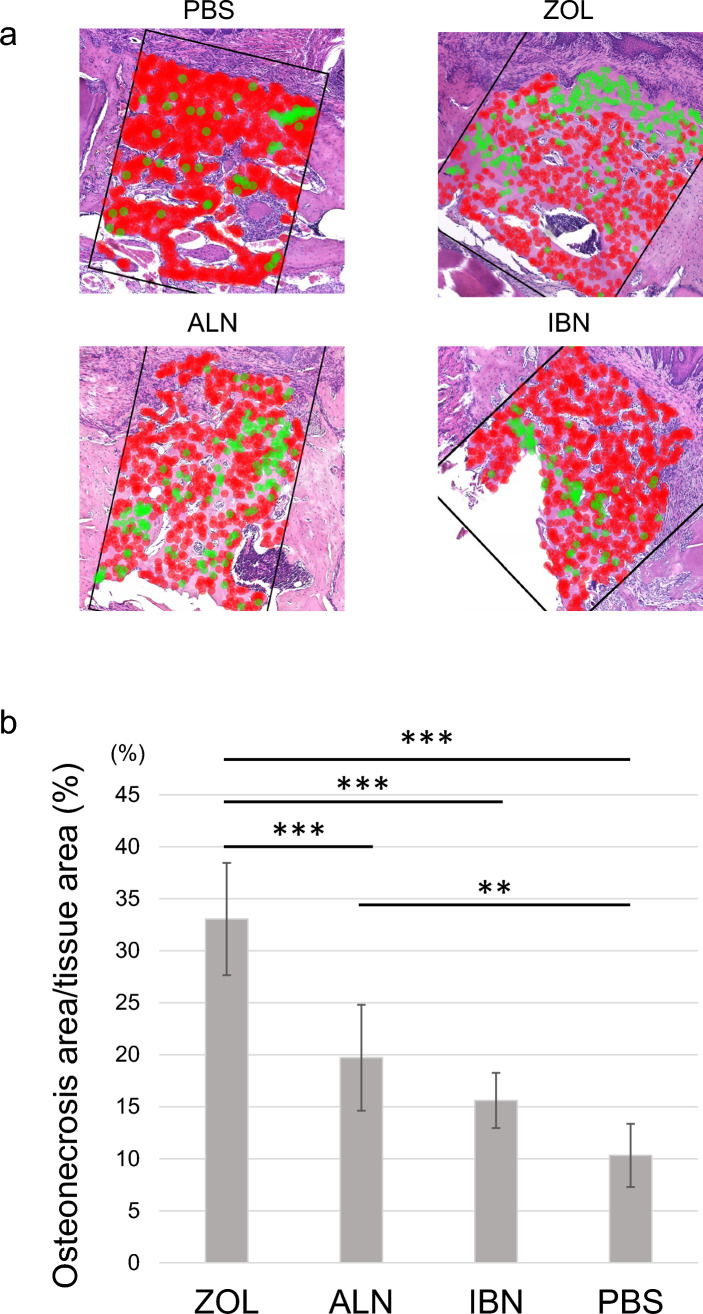
Zoledronate administration increases the extent of the osteonecrosis area relative to ibandronate or alendronate. **a** and **b** Eight-week-old female wild-type mice were administered zoledronate (ZOL), ibandronate (IBN), alendronate (ALN), or vehicle (PBS) (500 μg/kg/week each) once a week for two weeks and then the first molars in the mandible were extracted, as shown in Fig. 1. Mice were then administered ZOL, IBN, ALN or PBS once a week for 6 more weeks. Mice were sacrificed at 16-weeks, and mandibles were then removed, demineralized, paraffin-embedded, and sectioned for HE staining. The osteonecrosis area was calculated as a percentage of the area with empty lacunae among all lacunae. (a) Empty lacunae are indicated by green dots and non-empty lacunae by red dots. **b** Data represents mean percentage (%) of the osteonecrosis area ± SD (n = 7 ~ 10, *p < 0.05; **p < 0.01; ***p < 0.001; NS, not significant, based on one-way ANOVA and the Mann-Whitney test)

We next calculated the extent of the osteonecrosis area in jaw bones as a percentage of all bone area exhibiting empty lacunae (Fig. 3a and b). Based on this method, mice administered zoledronate showed significantly larger areas of osteonecrosis than did mice injected with ibandronate, alendronate or PBS (Fig. 3b).

### Zoledronate treatment increases bone mineral density more potently than alendronate or ibandronate

**Fig. 4:**
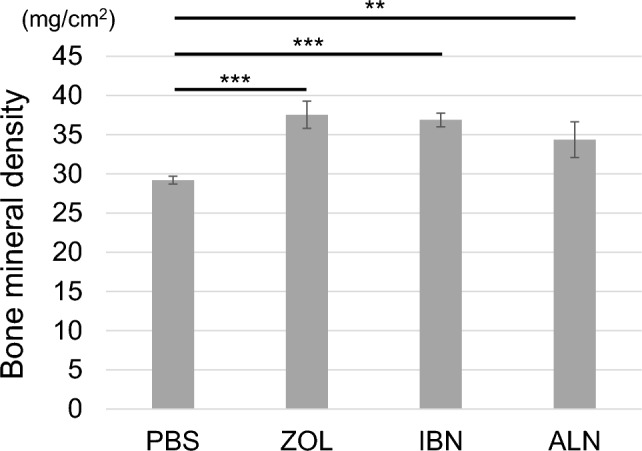
All three bisphosphonate treatments increase bone mineral density in mice. Mice administered zoledronate (ZOL), ibandronate (IBN), alendronate (ALN), or vehicle (PBS) (500 μg/kg/week each) as in Fig. 1a were euthanized at 16 weeks of age. Hindlimbs were then removed and bone mineral density quantified by DEXA. Shown is mean bone mineral density ± SD (n = 5, **p < 0.01; ***p < 0.001, based on one-way ANOVA)

Next, using the same mouse groups described above, we measured bone mineral density of the femur using Dual Energy X-ray Absorptiometry (DEXA) (Fig. 4). Bone mineral density showed a significant increase in the zoledronate, alendronate, or ibandronate groups relative to PBS controls (Fig. 4). Although mice administered zoledronate exhibited the highest bone mineral density, that increase was not significantly different from that seen in the alendronate and ibandronate groups (Fig. 4).

**Fig. 5:**
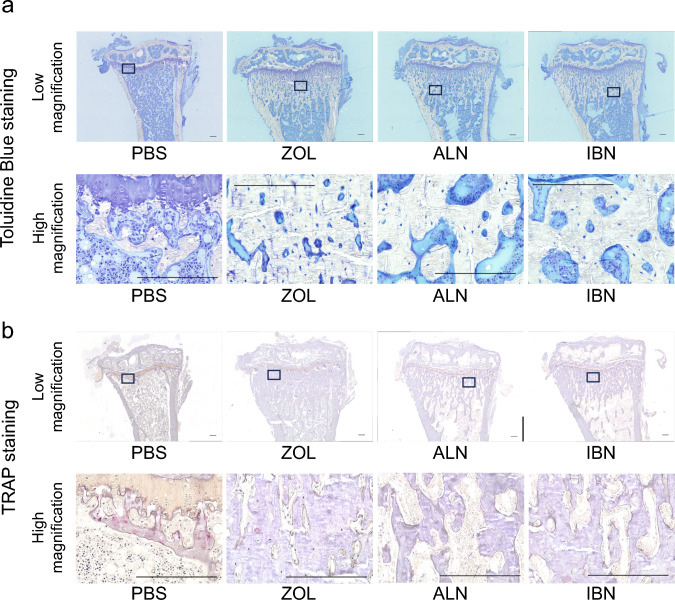
All three bisphosphonate treatments increase trabecular bone volume and inhibit osteoclast formation in mice. Mice administered zoledronate (ZOL), ibandronate (IBN), alendronate (ALN), or vehicle (PBS) (500 μg/kg/week each) as shown in Fig. 1a were euthanized at 16 weeks of age. Then, mouse tibias were collected, and non-decalcified specimens were prepared. Sections were stained with toluidine blue (**a**) or TRAP (**b**). Shown are low and high magnification images, as indicated. Scale bars = 200 μm

Next, we performed histological analysis using toluidine blue staining, which revealed increased bone mineralization at the epiphysis in either the zoledronate, alendronate, or ibandronate groups relative to the PBS control group (Fig. 5a). Tartrate-resistant acid phosphatase (TRAP) staining revealed few TRAP-positive osteoclasts in the epiphyseal region in either the zoledronate, alendronate, or ibandronate relative to the PBS groups, which showed significantly greater TRAP staining (Fig. 5b).

**Fig. 6:**
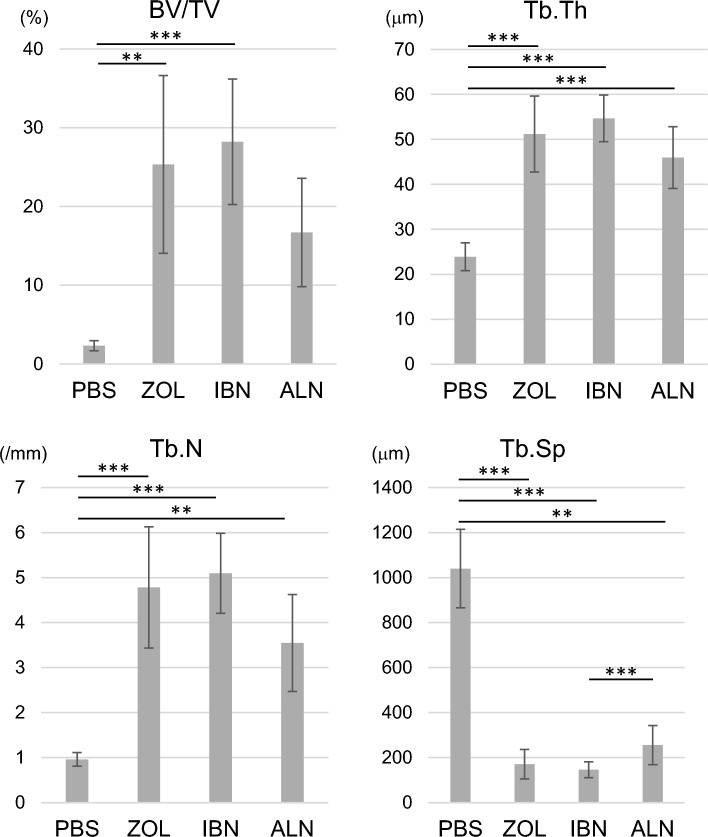
All three bisphosphonate treatments increase trabecular bone volume. Mice administered zoledronate (ZOL), ibandronate (IBN), alendronate (ALN), or vehicle (PBS) (500 μg/kg/week each) as shown in Fig. 1a were euthanized at 16 weeks of age. Then, mouse tibias were collected, and non-decalcified specimens were prepared. Sections were stained with toluidine blue, and bone morphometric analysis was performed to evaluate Bone Volume per Tissue Volume (BV/TV), Trabecular Thickness (Tb.Th), Trabecular Number (Tb.N) and Trabecular Separation (Tb.Sp). Data represents the mean indicated parameter ± SD (n = 5, **p < 0.01; ***p < 0.001; NS, not significant, based on one-way ANOVA)

Bone morphometric analysis revealed that relative to PBS treatment, administration of either zoledronate, ibandronate or alendronate significantly increased Trabecular Thickness (Tb.Th), and Trabecular Number (Tb.N), and significantly decreased Trabecular Separation (Tb.Sp) (Fig. 6). Also, we observed no significant differences among drugs tested in Tb.Th and Tb.N (Fig. 6). Bone Volume per Tissue Volume (BV/TV) was significantly elevated in zoledronate and ibandronate-treated mice relative to mice treated with PBS (Fig. 6).

**Fig. 7:**
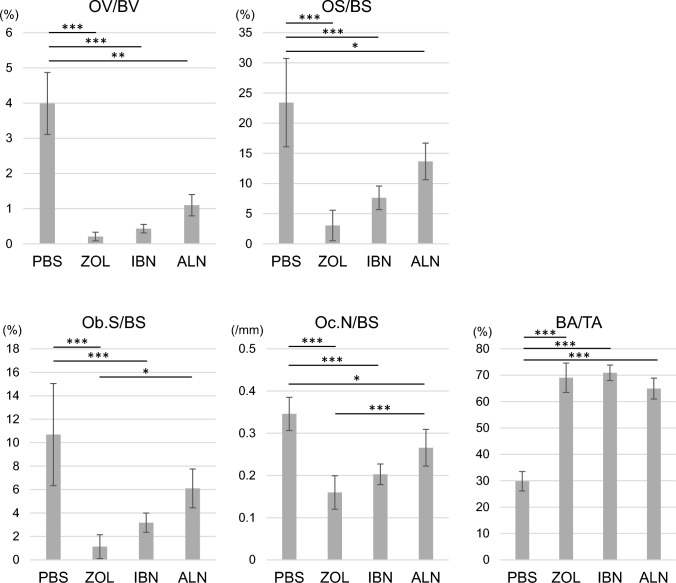
All three bisphosphonate treatments inhibit osteoclastogenesis and osteoblastogenesis in vivo. Mice administered zoledronate (ZOL), ibandronate (IBN), alendronate (ALN), or vehicle (PBS) (500 μg/kg/week each) as shown in Fig. 1a were euthanized at 16 weeks of age. Then, mouse tibias were collected, and non-decalcified specimens were prepared. Sections were stained with toluidine blue or TRAP, and bone morphometric analysis was performed to evaluate Osteoid Volume per Bone Volume (OV/BV), Osteoid Surface per Bone Surface (OS/BS), Osteoblast Surface per Bone Surface (Ob.S/BS), Osteoclast Number per Bone Surface (Oc.N/BS) and Bone Area per Tissue Area (BA/TA). Data represents the mean indicated parameter ± SD (n = 5, *p < 0.05; **p < 0.01; ***p < 0.001; NS, not significant, based on one-way ANOVA)

In addition, we observed significant increases in Bone Area per Tissue Area (BA/TA) and significant decreases in Osteoid Volume per Bone Volume (OV/BV), Osteoid Surface per Bone Surface (OS/BS) and Osteoclast Number per Bone surface (Oc.N/BS) in mice treated with either zoledronate, ibandronate or alendronate as compared to PBS (Fig. 7). Finally, osteoblast Surface per Bone Surface (Ob.S/BS) was significantly decreased by either zoledronate or ibandronate administration compared with PBS (Fig. 7).

### Zoledronate most potently inhibits osteoclastogenesis among bisphosphonates tested

**Fig. 8:**
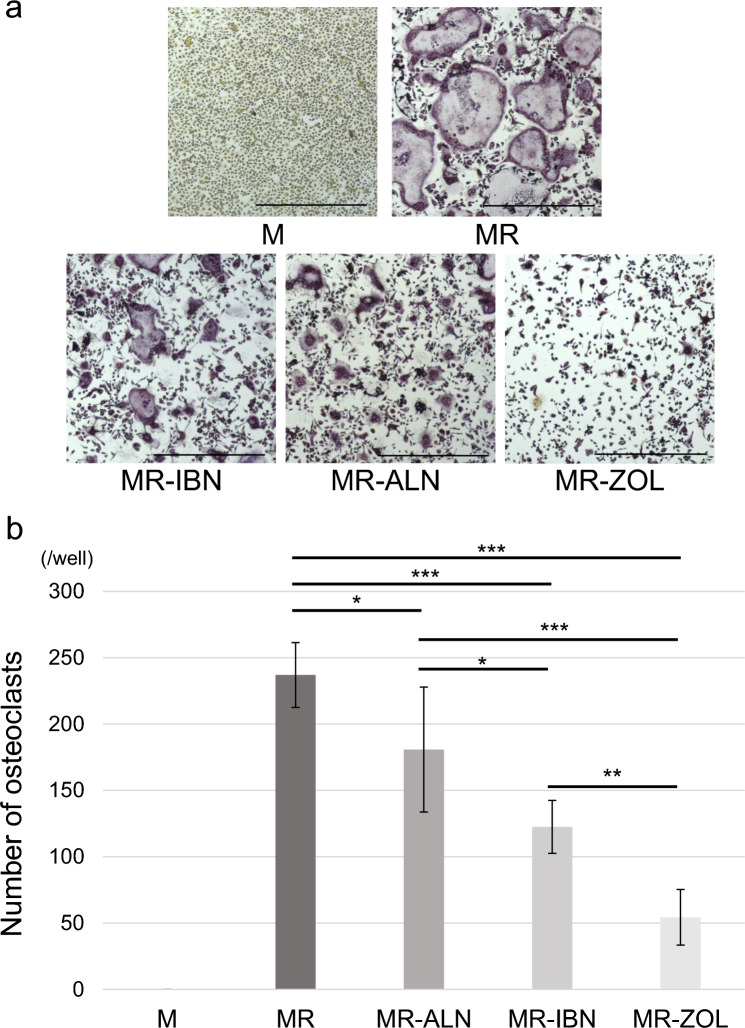
Zoledronate more potently inhibits osteoclastogenesis than does ibandronate or alendronate. Bone marrow cells were harvested from femur and tibia of wild-type mice and cultured 72 h in the presence of M-CSF. Adherent cells were then collected and cultured in medium containing M-CSF (M) or M plus RANKL (MR), with or without zoledronate (ZOL), ibandronate (IBN), and alendronate (ALN) (200 ng/ml each). Culture medium was replaced every two days. After 5 days of cultivation with BPs, cells were fixed and stained with TRAP. **a** Representative images showing TRAP staining. Scale bars = 200 μm. **b** Quantification ± SD of multi-nuclear TRAP-positive osteoclasts (defined as containing more than 10 nuclei) (n = 6, *p < 0.05; **p < 0.01; ***p < 0.001; NS, not significant, based on one-way ANOVA)

Finally, we evaluated effects of zoledronate, alendronate, or ibandronate on osteoclast differentiation in vitro. To do so we cultured osteoclast progenitor cells from wild-type mice with M-CSF alone or M-CSF + RANKL, with or without zoledronate, alendronate, or ibandronate, and then assessed osteoclast formation by TRAP staining (Fig. 8a). We defined osteoclasts as TRAP-positive cells containing 10 or more nuclei. Quantification of the number of osteoclasts per well showed that treatment with any one of the 3 BPs significantly inhibited formation of multi-nuclear TRAP-positive osteoclasts relative to the control (MCSF + RANKL group), but inhibition by zoledronate was significantly more potent than that mediated by the other two agents (Fig. 8b).

## Discussion

MRONJ prevention is critical, as MRONJ development significantly impairs patients’ ADL and quality of life. Here, we report that ONJ severity increased in mice in the presence of drugs that strongly inhibit osteoclastogenesis.

BPs have a basic “P-C-P structure” in which two phosphonic acid anion groups (phosphonates) are covalently bound to carbon [7, 29]. First-generation BPs such as etidronates lack nitrogen in the side chain, while third-generation risedronates and minodronates contain cyclic nitrogen in the side chain. Zoledronate is a third-generation BP, while second generation ibandronate and alendronate contain nitrogen in the side chain. Second and later generation BPs also show high affinity for hydroxyapatite and adsorb to the bone surface when absorbed in the body [7, 29]. When taken up by osteoclasts during bone resorption, bisphosphonates inhibit farnesyl pyrophosphate synthase (FPPS) activity and induce osteoclast apoptosis [30]. However, mechanisms underlying varying osteoclast apoptosis-inducing activity of second and later generation BPs have not been identified. Zoledronate is known to have the highest affinity for hydroxyapatite relative to all other BPs [7]. In fact, in this study, zoledronate showed a higher, although not significantly so, capacity to increase bone mineral density than ibandronate and alendronate at the same dose. On the other hand, zoledronate showed significantly stronger osteoclastogenesis inhibitory activity than ibandronate and alendronate in an in vitro osteoclast culture system established on cell culture dishes without hydroxyapatite.

Ibandronate and alendronate have been administered to osteoporotic patients orally or via intravenous injection (ibandronate) or intravenous drip (alendronate), and all approaches significantly increase bone mineral density and prevent fragility fractures [31–35]. Zoledronate is the only known drug that improves life expectancy as bone mineral density increases and fragility fractures decrease in osteoporotic patients [36, 37]. Systematic review of 27 studies including 5391 cases showed that intravenously administered bisphosphonates were associated with a higher incidence of MRONJ development than orally administered bisphosphonates [38]. In patients, zoledronate (4 mg) is administered via intravenous drip once a year, ibandronate (1 mg) via intravenous injection once a month, and alendronate (900 µg) by intravenous drip once every 4 weeks. This translates into a yearly dosage of 4 mg for zoledronate, 12 mg for ibandronate, and 11.7 mg for alendronate. Clinical trials of each of these drugs have been conducted using these dosages and administration routes. This means that the dose per period is the lowest for zoledronate among the drugs. Nevertheless, among bisphosphonates, MRONJ has been reported to be most likely to occur in patients treated with zoledronate. For example, in a randomized clinical trial metanalysis comparing the incidence of MRONJ in 5312 patients treated with ibandronate, risedronate, pamidronate, clodronate or zoledronate and 5382 patients who received placebo or no treatment, total 10,694 subjects, zoledronate was demonstrated significantly associated with MRONJ development among the bisphosphonates even though zoledronate is administered at the lowest dose per period [39]. Higher incidence of MRONJ development was also demonstrated in patients treated with zoledronate compared with those administered with other bisphosphonates by others [40]. However, it remained unclear why zoledronate, the lowest dose per period among the bisphosphonates, was associated with the highest risk of developing MRONJ. Indeed, there are no known clinical or animal studies that have compared MRONJ risk in patients treated with each of these drugs at the same dose and by the same method. In the present study, we found that zoledronate was most potent in increasing ONJ severity relative to ibandronate and alendronate in a mouse tooth extraction model in which zoledronate, ibandronate, or alendronate were administered at equivalent doses. In this study, we showed that zoledronate was the most effective inhibitor of osteoclasts among the bisphosphonates tested when compared at the same dose. Our data suggests that osteonecrosis development was significantly higher in mice treated with zoledronate than those administered other bisphosphonates. This information may be useful in providing information on the risk of MRONJ clinically. Since the patients receiving zoledronate are at higher risk of developing MRONJ than those receiving other bisphosphonates [40, 41], it is imperative to strictly manage oral hygiene and establish measures to prevent the onset of MRONJ. Since zoledronate is the only BP that can be administered as a bone-modifying agent to patients with metastatic bone tumors [42, 43], MRONJ should be closely evaluated in these patients also. Denosumab, a neutralizing antibody targeting RANKL, a cytokine essential for osteoclast differentiation, is also used as a bone modifying agent [5, 6, 44, 45], and denosumab reportedly carries the risk of MRONJ similar to zoledronate [46].

Various animal models have been introduced to mimic MRONJ pathophysiology, but most involve extraction of teeth in the presence of strong bone resorption inhibitors [47]. ONJ induction rate varies from model to model: some models employ administration of additional agents such as LPS to potentiate ONJ induction [48]. In any case, it is difficult to completely reproduce MRONJ seen in humans in a mouse model, which is an experimental limitation. In this study, we employed a model in which teeth are extracted under BP administration [18, 19]. In this model, ONJ and bone palpable fistula formation occurs, and ONJ incidence is significantly exacerbated by zoledronate administration. Thus, we consider this to be a MRONJ model, at least in part [1]. Clinically, zoledronate and alendronate are administered via infusion, while ibandronate is administered intravenously; however, given the technical difficulties administering drugs to mice via infusion, in this study all agents were administered subcutaneously. Here, we compared effects of ibandronate and alendronate with those of zoledronate in this model and found that zoledronate increased ONJ risk more potently than did ibandronate and alendronate at the same dose.

We demonstrated that zoledronate was more potent in inducing MRONJ development than either ibandronate or alendronate (Figs. 2 and 3) and observed no significant differences in bone mineral density increases or bone parameters based on bone morphometric analysis among the three agents (Figs. 4, 5, 6, 7). These findings suggest that the effect of these drugs in vivo on bone parameters may have already reached a plateau and that factors other than osteoclasts or possibly activity of other cell types may promote differences in MRONJ development seen among agents tested. Indeed, bisphosphonates, especially amino-bisphosphonates (N-BPs), have been shown to have a variety of effects on cells other than osteoclasts. Moreover, zoledronate reportedly exhibits anti-angiogenic effects [49, 50]. while alendronate and zoledronate have direct tumoricidal activity as well as immune modulatory effects on myeloid and T cells in vitro and in animal models of cancer [51]. Thus, it is possible that those effects, rather than inhibition of bone resorption, may underlie induction of osteonecrosis of the jaw by N-BPs. On the other hand, it is not clear whether N-BPs can exert systemic effects, since they are rapidly cleared by the kidney and strongly adhere to hydroxyapatite in bone [7]. Indeed, there are no reports of adverse events on vascular endothelial cells in humans by zoledronate administration. Moreover, systemic osteonecrosis has not been reported following administration of BPs such as zoledronate. In our mouse model we also demonstrated that formation of empty lacunae was comparable to that seen in PBS-treated control mice, even when BPs were administered on the control side without tooth extraction (Fig. 2).

While changes in bone mineral density and bone morphometric parameters often require time to occur after BP administration, acute-phase reactions after BP administration have occasionally been reported to occur soon after administration [52]. Such an acute reaction accompanied by flu-like symptoms of fever and joint pain has reportedly occurred after zoledronate administration [53]. Also, following inhibition of osteoclast differentiation, osteoclast progenitor cells have been shown to differentiate into inflammatory cytokine-producing macrophage lineage cells [18]. Stronger inhibition of osteoclast differentiation likely results in induction of higher levels of inflammatory cytokines, which are known risks for osteonecrosis development [17]. We have shown that among BPs tested, zoledronate was the most potent inhibitor of osteoclastogenesis in vitro, which may promote induction of inflammatory cytokines and account for why zoledronate induces MRONJ development more potently than ibandronate or alendronate. These observations, however, do not preclude the need to consider other potential causes of jaw osteonecrosis in order to precisely define mechanisms underlying MRONJ development after BP administration.

Our study also shows that use of strong bone resorption inhibitors requires special efforts to prevent ONJ onset. Collaboration between medical and dental professionals could be useful in preventing MRONJ. Moreover, particular consideration is required when drugs with strong osteoclastogenic potential are administered to patients.

## Materials and methods

### ONJ model mice

C57BL/6 J wild-type mice used in the study were purchased from SLC (Shizuoka, Japan). All animals were maintained under specific pathogen-free conditions, and were housed under a 12-h dark–light cycle (light from 07:00 to 19:00) at 22 ± 1 °C with ad libitum food and water. The Animal Care and Use Committee of Kumamoto University approved the protocols for animal experiments.

Based on Soma et al. [4], we generated ONJ model mice by administering subcutaneously zoledronate (Zometa®, designated ZOL; Novartis, Stein, Switzerland), ibandronate (Bonviva®, designated IBN; Taisho Pharmaceutical, Tokyo, Japan), or alendronate (Bonalon®, designated ALN; Teijin Pharma, Tokyo, Japan) to 8-week-old wild-type female mice. Control mice were injected with PBS vehicle. All drugs were administered weekly at 500 μg/kg/week. Two weeks later, the right first molar was extracted. Before tooth extraction, all mice received a mixture of medetomidine hydrochloride (0.3 mg/kg), midazolam (4 mg/kg) and butorphanol tartrate (5 mg/kg) anesthesia by intraperitoneal injection. BP or vehicle injections were continued for 6 weeks weekly after tooth extraction, and mice were euthanized at 16-weeks of age.

### Histopathological analysis

Mouse mandibles were removed, decalcified in Morse’s solution (Fujifilm Wako Pure Chemical Corporation, Osaka, Japan), and paraffin-embedded. Tissue from the tooth extraction site was cut in sagittal sections, and hematoxylin-eosin (HE) staining was performed using general procedures. Loss of cells from bone lacunae was defined as an empty lacuna [54]. The osteonecrosis area in the jaw bones was calculated as the percentage of area with empty lacunae in all bone area. One extracted tooth was set as the region of interest (ROI), and the percentage of empty lacunae among all lacunae was calculated (Fig. 1c and d).

### Analysis of skeletal morphology

Hindlimbs of 16-week-old mice that had been euthanized after ZOL, IBN, ALE, or vehicle administration were collected and fixed in 70% ethanol. Following fixation, bone mineral density was measured using DEXA as described [55]. Specimens were also embedded in glycolmethacrylate resin blocks to create non-decalcified specimens. Toluidine blue and TRAP stainings were performed as described [56].

### In vitro osteoclast formation

Bone marrow cells were harvested from the femur and tibia of mice and cultured 72 h in medium containing 10% fetal bovine serum (FBS), GlutaMAX (Thermo Fisher Scientific, Waltham, MA, USA), and M-CSF (R&D Systems, Minneapolis, MN, USA; 50 ng/ml). Adherent cells were then collected and cultured in a 96-well plate at 10^5^ cells per well in the presence of M-CSF with or without RANKL (25 ng/ml). Some cells were also treated with ZOL, IBN or ALN (200 ng/ml each). The culture medium was replaced every two days. Following a five-day culture period, adherent cells were fixed in 1% formalin, and stained with TRAP (Sigma-Aldrich, St. Louis, MO, USA). Formation of multi-nuclear TRAP-positive osteoclasts was evaluated under a microscope.

### Statistical analysis

All statistical analyses were performed with EZR (Saitama Medical Center, Jichi Medical University, Saitama, Japan) [57], a graphical user interface for R (The R Foundation for Statistical Computing, Vienna, Austria). Specifically, we used a modified version of R commander designed to add statistical functions frequently used in biostatistics. Quantitative data were expressed as means ± SD. Statistical significance was assessed using Student’s t-test, the Mann–Whitney test or one-way analysis of variance (ANOVA). (*p < 0.05; **p < 0.01; ***p < 0.001; NS, not significant).

## Data Availability

The datasets used and/or analyzed in the current study are available from the corresponding author on reasonable request.

## References

[1] Ruggiero SL, Dodson TB, Aghaloo T, Carlson ER, Ward BB, Kademani D (2022) American Association of Oral and Maxillofacial Surgeons’ Position Paper on Medication-Related Osteonecrosis of the Jaws-2022 Update (in Eng). J Oral Maxillofac Surg 80:920–943. 10.1016/j.joms.2022.02.008 35300956 10.1016/j.joms.2022.02.008

[2] Marx RE (2003) Pamidronate (Aredia) and zoledronate (Zometa) induced avascular necrosis of the jaws: a growing epidemic. J Oral Maxillofac Surg 61:1115–1117. 10.1016/s0278-2391(03)00720-1 12966493 10.1016/s0278-2391(03)00720-1

[3] Beth-Tasdogan NH, Mayer B, Hussein H, Zolk O, Peter JU (2022) Interventions for managing medication-related osteonecrosis of the jaw (in Eng). Cochrane Database Syst Rev. 10.1002/14651858.CD012432.pub3 35866376 10.1002/14651858.CD012432.pub3 PMC9309005

[4] Disel U, Besen AA, Özyi̇lkan Ö, Er E, Canpolat T (2012) A case report of bevacizumab-related osteonecrosis of the jaw: old problem, new culprit. Oral Oncol 10.1016/j.oraloncology.2011.07.030 10.1016/j.oraloncology.2011.07.030 21865077

[5] Fizazi K, Lipton A, Mariette X, Body JJ, Rahim Y, Gralow JR, Gao G, Wu L, Sohn W, Jun S (2009) Randomized Phase II Trial of denosumab in patients with bone metastases from prostate cancer, breast cancer, or other neoplasms after intravenous bisphosphonates (in Eng). J Clin Oncol 27:1564–1571. 10.1200/jco.2008.19.2146 19237632 10.1200/JCO.2008.19.2146

[6] Ford J, Cummins E, Sharma P, Elders A, Stewart F, Johnston R, Royle P, Jones R, Mulatero C, Todd R, Mowatt G (2013) Systematic review of the clinical effectiveness and cost-effectiveness, and economic evaluation, of denosumab for the treatment of bone metastases from solid tumours (in Eng). Health Technol Assess 17:1–386. 10.3310/hta17290 23870108 10.3310/hta17290 PMC4780939

[7] Russell RG, Watts NB, Ebetino FH, Rogers MJ (2008) Mechanisms of action of bisphosphonates: similarities and differences and their potential influence on clinical efficacy (in Eng). Osteoporos Int (Osteoporos Int) 19:733–759. 10.1007/s00198-007-0540-8 18214569 10.1007/s00198-007-0540-8

[8] Allen MR (2009) Bisphosphonates and osteonecrosis of the jaw: moving from the bedside to the bench (in Eng). Cells Tissues Organs 189:289–294. 10.1159/000151371 18698128 10.1159/000151371 PMC2824205

[9] Baron R, Ferrari S, Russell RGG (2011) Denosumab and bisphosphonates: different mechanisms of action and effects. Bone 48:677–692. 10.1016/j.bone.2010.11.020 21145999 10.1016/j.bone.2010.11.020

[10] Pimolbutr K, Porter S, Fedele S (2018) Osteonecrosis of the jaw associated with antiangiogenics in antiresorptive-naïve patient: a comprehensive review of the literature. Biomed Res Int 2018:8071579. 10.1155/2018/8071579 29850569 10.1155/2018/8071579 PMC5937620

[11] Nicolatou-Galitis O, Kouri M, Papadopoulou E, Vardas E, Galiti D, Epstein JB, Elad S, Campisi G, Tsoukalas N, Bektas-Kayhan K, Tan W, Body J-J, Migliorati C, Lalla RV, for the MBSG (2019) Osteonecrosis of the jaw related to non-antiresorptive medications: a systematic review. Support Care Cancer 27:383–394. 10.1007/s00520-018-4501-x 30353228 10.1007/s00520-018-4501-x

[12] Aljohani S, Fliefel R, Ihbe J, Kühnisch J, Ehrenfeld M, Otto S (2017) What is the effect of anti-resorptive drugs (ARDs) on the development of medication-related osteonecrosis of the jaw (MRONJ) in osteoporosis patients: a systematic review. J Cranio-Maxillofac Surg 45:1493–1502. 10.1016/j.jcms.2017.05.028 10.1016/j.jcms.2017.05.028 28687467

[13] Peer A, Khamaisi M (2015) Diabetes as a risk factor for medication-related osteonecrosis of the jaw. J Dent Res 94:252–260. 10.1177/0022034514560768 25477311 10.1177/0022034514560768 PMC4438733

[14] Park S-M, Lee J-H (2022) Effects of Type 2 Diabetes mellitus on osteoclast differentiation, activity, and cortical bone formation in POSTmenopausal MRONJ patients. J Clin Med 11:2377 35566506 10.3390/jcm11092377 PMC9102751

[15] Kumar SK, Gorur A, Schaudinn C, Shuler CF, Costerton JW, Sedghizadeh PP (2010) The role of microbial biofilms in osteonecrosis of the jaw associated with bisphosphonate therapy (in Eng). Curr Osteoporos Rep 8:40–48. 10.1007/s11914-010-0008-1 20425090 10.1007/s11914-010-0008-1

[16] Otto S, Aljohani S, Fliefel R, Ecke S, Ristow O, Burian E, Troeltzsch M, Pautke C, Ehrenfeld M (2021) Infection as an important factor in medication-related osteonecrosis of the jaw (MRONJ). Medicina 57:463 34065104 10.3390/medicina57050463 PMC8151678

[17] Morita M, Iwasaki R, Sato Y, Kobayashi T, Watanabe R, Oike T, Nakamura S, Keneko Y, Miyamoto K, Ishihara K, Iwakura Y, Ishii K, Matsumoto M, Nakamura M, Kawana H, Nakagawa T, Miyamoto T (2017) Elevation of pro-inflammatory cytokine levels following anti-resorptive drug treatment is required for osteonecrosis development in infectious osteomyelitis (in Eng). Sci Rep 7:46322. 10.1038/srep46322 28387378 10.1038/srep46322 PMC5384218

[18] Soma T, Iwasaki R, Sato Y, Kobayashi T, Nakamura S, Kaneko Y, Ito E, Okada H, Watanabe H, Miyamoto K, Matsumoto M, Nakamura M, Asoda S, Kawana H, Nakagawa T, Miyamoto T (2021) Tooth extraction in mice administered zoledronate increases inflammatory cytokine levels and promotes osteonecrosis of the jaw (in Eng). J Bone Miner Metab 39:372–384. 10.1007/s00774-020-01174-2 33200254 10.1007/s00774-020-01174-2

[19] Soma T, Iwasaki R, Sato Y, Kobayashi T, Ito E, Matsumoto T, Kimura A, Miyamoto K, Matsumoto M, Nakamura M, Morita M, Asoda S, Kawana H, Nakagawa T, Miyamoto T (2022) Osteonecrosis development by tooth extraction in zoledronate treated mice is inhibited by active vitamin D analogues, anti-inflammatory agents or antibiotics (in Eng). Sci Rep 12:19. 10.1038/s41598-021-03966-6 34997043 10.1038/s41598-021-03966-6 PMC8742126

[20] Li Q, Pu Y, Lu H, Zhao N, Wang Y, Guo Y, Guo C (2021) Porphyromonas, Treponema, and Mogibacterium promote IL8/IFNγ/TNFα-based pro-inflammation in patients with medication-related osteonecrosis of the jaw. J Oral Microbiol 13:1851112. 10.1080/20002297.2020.1851112 10.1080/20002297.2020.1851112 PMC7717612 33391627

[21] Hagino H, Jackson M, Gitlin M, Wessler Z (2021) Estimating the future clinical and economic benefits of improving osteoporosis diagnosis and treatment among women in Japan: a simulation projection model from 2020 to 2040. Arch Osteoporos 16:156. 10.1007/s11657-021-01019-z 34642839 10.1007/s11657-021-01019-z

[22] LeBoff MS, Greenspan SL, Insogna KL, Lewiecki EM, Saag KG, Singer AJ, Siris ES (2022) Correction to: The clinician’s guide to prevention and treatment of osteoporosis. Osteoporos Int 33:2243–2343. 10.1007/s00198-022-06479-8 35900384 10.1007/s00198-022-06479-8 PMC9546943

[23] Ezra A, Golomb G (2000) Administration routes and delivery systems of bisphosphonates for the treatment of bone resorption. Adv Drug Deliv Rev 42:175–195. 10.1016/S0169-409X(00)00061-2 10963835 10.1016/s0169-409x(00)00061-2

[24] Asagiri M, Takayanagi H (2007) The molecular understanding of osteoclast differentiation. Bone 40:251–264. 10.1016/j.bone.2006.09.023 17098490 10.1016/j.bone.2006.09.023

[25] Takahashi N, Akatsu T, Udagawa N, Sasaki T, Yamaguchi A, Moseley JM, Martin TJ, Suda T (1988) Osteoblastic cells are involved in osteoclast formation. Endocrinology 123:2600–2602. 10.1210/endo-123-5-2600 2844518 10.1210/endo-123-5-2600

[26] Lacey DL, Timms E, Tan HL, Kelley MJ, Dunstan CR et al (1998) Osteoprotegerin ligand is a cytokine that regulates osteoclast differentiation and activation. Cell 93:165–176. 10.1016/S0092-8674(00)81569-X 9568710 10.1016/s0092-8674(00)81569-x

[27] Kim H-W, Lee M-W, Lee J-H, Kim M-Y (2021) Comparison of the effect of oral versus intravenous bisphosphonate administration on osteoclastogenesis in advanced-stage medication-related osteonecrosis of the jaw patients. J Clin Med 10:2988 34279472 10.3390/jcm10132988 PMC8268194

[28] Lee S-H, Choi S-Y, Bae M-S, Kwon T-G (2021) Characteristics of patients with osteonecrosis of the jaw with oral versus intravenous bisphosphonate treatment. Maxillofac Plastic Reconstr Surg 43:24. 10.1186/s40902-021-00310-w 10.1186/s40902-021-00310-w PMC8266939 34236538

[29] Russell RG (2006) Bisphosphonates: from bench to bedside (in Eng). Ann N Y Acad Sci 1068:367–401. 10.1196/annals.1346.041 16831938 10.1196/annals.1346.041

[30] Dunford JE, Thompson K, Coxon FP, Luckman SP, Hahn FM, Poulter CD, Ebetino FH, Rogers MJ (2001) Structure-activity relationships for inhibition of farnesyl diphosphate synthase in vitro and inhibition of bone resorption in vivo by nitrogen-containing bisphosphonates. J Pharmacol Exp Ther 296:235–242 11160603

[31] Liberman UA, Weiss SR, Bröll J, Minne HW, Quan H, Bell NH, Rodriguez-Portales J, Downs RW, Dequeker J, Favus M, Seeman E, Recker RR, Capizzi T, Santora AC, Lombardi A, Shah RV, Hirsch LJ, Karpf DB (1995) Effect of oral alendronate on bone mineral density and the incidence of fractures in postmenopausal osteoporosis. N Engl J Med 333:1437–1444. 10.1056/NEJM199511303332201 7477143 10.1056/NEJM199511303332201

[32] Thiébaud D, Burckhardt P, Kriegbaum H, Huss H, Mulder H, Juttmann JR, Schöter KH (1997) Three monthly intravenous injections of ibandronate in the treatment of postmenopausal osteoporosis. Am J Med 103:298–307. 10.1016/S0002-9343(97)00249-0 9382122 10.1016/s0002-9343(97)00249-0

[33] Felsenberg D, Miller P, Armbrecht G, Wilson K, Schimmer RC, Papapoulos SE (2005) Oral ibandronate significantly reduces the risk of vertebral fractures of greater severity after 1, 2, and 3 years in postmenopausal women with osteoporosis. Bone 37:651–654. 10.1016/j.bone.2005.05.004 16126016 10.1016/j.bone.2005.05.004

[34] Wells GA, Cranney A, Peterson J, Boucher M, Shea B, Welch V, Coyle D, Tugwell P (2008) Alendronate for the primary and secondary prevention of osteoporotic fractures in postmenopausal women. Cochrane Database Syst Rev. 10.1002/14651858.CD001155.pub2 18253985 10.1002/14651858.CD001155.pub2

[35] Eisman JA, Civitelli R, Adami S, Czerwiński E, Recknor C, Prince RL, Reginster J-Y, Zaidi M, Felsenberg D, Hughes C, Mairon N, Masanauskaite D, Reid DM, Delmas PD, Recker RR (2008) Efficacy and tolerability of intravenous ibandronate injections in postmenopausal osteoporosis: 2-year results from the DIVA study. J Rheumatol 35:488–497 18260172

[36] Reid IR, Brown JP, Burckhardt P, Horowitz Z, Richardson P et al (2002) Intravenous zoledronic acid in postmenopausal women with low bone mineral density. N Engl J Med 346:653–661. 10.1056/NEJMoa011807 11870242 10.1056/NEJMoa011807

[37] Black DM, Delmas PD, Eastell R, Reid IR, Boonen S et al (2007) Once-yearly zoledronic acid for treatment of postmenopausal osteoporosis. N Engl J Med 356:1809–1822. 10.1056/NEJMoa067312 17476007 10.1056/NEJMoa067312

[38] Martins LHI, Ferreira DC, Silva MT, Motta RHL, Franquez RT, Bergamaschi CdC (2023) Frequency of osteonecrosis in bisphosphonate users submitted to dental procedures: a systematic review. Oral Dis 29:75–99. 10.1111/odi.14003 34402147 10.1111/odi.14003

[39] Mauri D, Valachis A, Polyzos IP, Polyzos NP, Kamposioras K, Pesce LL (2009) Osteonecrosis of the jaw and use of bisphosphonates in adjuvant breast cancer treatment: a metanalysis. Breast Cancer Res Treat 116:433–439. 10.1007/s10549-009-0432-z 19521766 10.1007/s10549-009-0432-z

[40] Amigues C, Fresse A, Roux CH, Gauthier S, Vieillard M-H, Drici M-D, Breuil V (2023) Zoledronate and osteonecrosis of the jaw in osteoporosis: incidence and risk factors. Analysis of the French Pharmacovigilance Database. Joint Bone Spine 90:105599. 10.1016/j.jbspin.2023.105599 37271278 10.1016/j.jbspin.2023.105599

[41] Veszelyné Kotán E, Bartha-Lieb T, Parisek Z, Meskó A, Vaszilkó M, Hankó B (2019) Database analysis of the risk factors of bisphosphonate-related osteonecrosis of the jaw in Hungarian patients. BMJ Open 9:e025600. 10.1136/bmjopen-2018-025600 31122970 10.1136/bmjopen-2018-025600 PMC6537976

[42] Rosen LS, Gordon D, Tchekmedyian S, Yanagihara R, Hirsh V, Krzakowski M, Pawlicki M, Souza Pd, Zheng M, Urbanowitz G, Reitsma D, Seaman JJ (2003) Zoledronic acid versus placebo in the treatment of skeletal metastases in patients with lung cancer and other solid tumors: a Phase III, double-blind, randomized trial—the zoledronic acid lung cancer and other solid tumors Study Group. J Clin Oncol 21:3150–3157. 10.1200/jco.2003.04.105 12915606 10.1200/JCO.2003.04.105

[43] Kohno N, Aogi K, Minami H, Nakamura S, Asaga T, Iino Y, Watanabe T, Goessl C, Ohashi Y, Takashima S (2005) Zoledronic acid significantly reduces skeletal complications compared with placebo in japanese women with bone metastases from breast cancer: a randomized, placebo-controlled trial. J Clin Oncol 23:3314–3321. 10.1200/jco.2005.05.116 15738536 10.1200/JCO.2005.05.116

[44] Bone HG, Wagman RB, Brandi ML, Brown JP, Chapurlat R et al (2017) 10 years of denosumab treatment in postmenopausal women with osteoporosis: results from the Phase 3 Randomised FREEDOM trial and open-label extension. Lancet Diabetes Endocrinol 5:513–523. 10.1016/S2213-8587(17)30138-9 28546097 10.1016/S2213-8587(17)30138-9

[45] Smith MR, Saad F, Coleman R, Shore N, Fizazi K et al (2012) Denosumab and bone-metastasis-free survival in men with castration-resistant prostate cancer: results of a Phase 3, Randomised, placebo-controlled trial. The Lancet 379:39–46. 10.1016/S0140-6736(11)61226-9 10.1016/S0140-6736(11)61226-9 PMC3671878 22093187

[46] Diz P, López-Cedrún JL, Arenaz J, Scully C (2012) Denosumab-related osteonecrosis of the jaw. J Am Dental Assoc 143:981–984 10.14219/jada.archive.2012.0323 10.14219/jada.archive.2012.0323 22942143

[47] Aguirre JI, Castillo EJ, Kimmel DB (2021) Preclinical models of medication-related osteonecrosis of the jaw (MRONJ) (in Eng). Bone 153:116184. 10.1016/j.bone.2021.116184 34520898 10.1016/j.bone.2021.116184 PMC8743993

[48] Sakaguchi O, Kokuryo S, Tsurushima H, Tanaka J, Habu M, Uehara M, Nishihara T, Tominaga K (2015) Lipopolysaccharide aggravates bisphosphonate-induced osteonecrosis in rats. Int J Oral Maxillofac Surg 44:528–534. 10.1016/j.ijom.2014.08.011 25442743 10.1016/j.ijom.2014.08.011

[49] Wood J, Bonjean K, Ruetz S, Bellahcène A, Devy L, Foidart JM, Castronovo V, Green JR (2002) Novel antiangiogenic effects of the bisphosphonate compound zoledronic acid. J Pharmacol Exp Ther 302:1055–1061. 10.1124/jpet.102.035295 12183663 10.1124/jpet.102.035295

[50] Giraudo E, Inoue M, Hanahan D (2004) An amino-bisphosphonate targets MMP-9 - Expressing macrophages and angiogenesis to impair cervical carcinogenesis. J Clin Investig 114:623–633. 10.1172/JCI200422087 15343380 10.1172/JCI22087 PMC514591

[51] La-Beck NM, Liu X, Shmeeda H, Shudde C, Gabizon AA (2021) Repurposing amino-bisphosphonates by liposome formulation for a new role in cancer treatment. Semin Cancer Biol 68:175–185. 10.1016/j.semcancer.2019.12.001 31874280 10.1016/j.semcancer.2019.12.001

[52] Adami S, Bhalla AK, Dorizzi R, Montesanti F, Rosini S, Salvagno G, Lo Cascio V (1987) The acute-phase response after bisphosphonate administration. Calcif Tissue Int 41:326–331. 10.1007/BF02556671 3124942 10.1007/BF02556671

[53] Popp AW, Senn R, Curkovic I, Senn C, Buffat H, Popp PF, Lippuner K (2017) Factors associated with acute-phase response of bisphosphonate-naïve or pretreated women with osteoporosis receiving an intravenous first dose of zoledronate or ibandronate. Osteoporos Int 28:1995–2002. 10.1007/s00198-017-3992-5 28299378 10.1007/s00198-017-3992-5

[54] Fondi C, Franchi A (2007) Definition of bone necrosis by the pathologist (in Eng). Clin Cases Miner Bone Metab 4:21–26 22460748 PMC2781178

[55] Arima T, Sugimoto K, Taniwaki T, Maeda K, Shibata Y et al (2024) Cartilage tissues regulate systemic aging via ectonucleotide pyrophosphatase/phosphodiesterase 1 in mice. J Biol Chem 300:105512. 10.1016/j.jbc.2023.105512 38042486 10.1016/j.jbc.2023.105512 PMC10777000

[56] Ito E, Sato Y, Kobayashi T, Nakamura S, Kaneko Y, Soma T, Matsumoto T, Kimura A, Miyamoto K, Matsumoto H, Matsumoto M, Nakamura M, Sato K, Miyamoto T (2021) Food restriction reduces cortical bone mass and serum insulin-like growth factor-1 levels and promotes uterine atrophy in mice. Biochem Biophys Res Commun 534:165–171. 10.1016/j.bbrc.2020.11.122 33288195 10.1016/j.bbrc.2020.11.122

[57] Kanda Y (2013) Investigation of the freely available easy-to-use software ‘EZR’ for medical statistics. Bone Marrow Transplant 48:452–458. 10.1038/bmt.2012.244 23208313 10.1038/bmt.2012.244 PMC3590441

